# GEMS: A Fully Integrated PETSc-Based Solver for Coupled Cardiac Electromechanics and Bidomain Simulations

**DOI:** 10.3389/fphys.2018.01431

**Published:** 2018-10-16

**Authors:** Sander Arens, Hans Dierckx, Alexander V. Panfilov

**Affiliations:** ^1^Department of Physics and Astronomy, Ghent University, Ghent, Belgium; ^2^Laboratory of Computational Biology and Medicine, Ural Federal University, Ekaterinburg, Russia

**Keywords:** cardiac arrhythmias, electromechanics, cardiac modeling, ionic models, anatomical models

## Abstract

Cardiac contraction is coordinated by a wave of electrical excitation which propagates through the heart. Combined modeling of electrical and mechanical function of the heart provides the most comprehensive description of cardiac function and is one of the latest trends in cardiac research. The effective numerical modeling of cardiac electromechanics remains a challenge, due to the stiffness of the electrical equations and the global coupling in the mechanical problem. Here we present a short review of the inherent assumptions made when deriving the electromechanical equations, including a general representation for deformation-dependent conduction tensors obeying orthotropic symmetry, and then present an implicit-explicit time-stepping approach that is tailored to solving the cardiac mono- or bidomain equations coupled to electromechanics of the cardiac wall. Our approach allows to find numerical solutions of the electromechanics equations using stable and higher order time integration. Our methods are implemented in a monolithic finite element code GEMS (Ghent Electromechanics Solver) using the PETSc library that is inherently parallelized for use on high-performance computing infrastructure. We tested GEMS on standard benchmark computations and discuss further development of our software.

## 1. Introduction

The heart is an electromechanical pump whose mechanical contraction is initiated by electrical activation, in a process called excitation-contraction coupling. In normal circumstances, contraction is highly synchronized, resulting in an efficient throughput of oxygenated blood to the body. Failure in doing so can lead to sudden cardiac death. The contraction also affects excitation via the process called mechano-electrical feedback. An example of mechano-electrical feedback that has fatal consequences is *commotio cordis* (Maron and Estes, 2010), a long-known (Akenside, 1763; Meola, 1879; Nesbitt et al., 2001) phenomenon where a blow to the chest (even without damaging the heart) may cause ventricular fibrillation. *Commotio cordis* is still an important cause of sudden cardiac death in young athletes (Maron, 2003). The underlying mechanism of mechano-electrical feedback is caused by several factors, including stretch-activated ionic channels (Kohl et al., 2001). Although much is already known about the subcellular contributions to mechano-electrical feedback (Quinn et al., 2014), it is still unclear how these translate to macroscopic scales. Computational models can further help understand the mechanisms and consequences of cardiac mechano-electrical feedback up to the organ level.

The heart is mostly modeled as a continuum via partial differential equations (PDEs). For the spatial coupling between cells, the cardiac mono- or bidomain equations (Keener and Sneyd, 2009) are commonly used, in which any specific model for individual cardiac cells can be inserted. For the mechanical problem, the most commonly used are the PDEs of finite (hyper)elasticity (Nash and Hunter, 2000). The joint solution of these equations is a considerable numerical challenge. The difficulties largely originate from the different physical interactions that occur on a wide range of spatial and temporal scales (Plank et al., 2008; Keyes et al., 2013). The multiphysics nature makes it impossible to use a general-purpose black-box solver for this task. Solvers can only be optimal if they use as much information as possible about the problem. For example, implicit/explicit integrators need to know which processes are fast or slow, field-split preconditioners (Brown et al., 2012; Liu and Keyes, 2015) need to be able to extract fields belonging to different physics, and multigrid (Briggs et al., 2000; Trottenberg et al., 2000) and domain decomposition (Quarteroni and Valli, 1999; Smith et al., 2004) solvers need information about the meshes and discretizations.

In recent years, computational modeling of cardiac electromechanics has become an active field of research see e.g., (Göktepe and Kuhl, 2010; Lafortune et al., 2012; Land et al., 2012; Fritz et al., 2014; Rossi et al., 2014; Franzone et al., 2015; Augustin et al., 2016). However, different groups often use different descriptions for the same problems with different forms for deformation-dependent conduction tensors and sometimes convective terms in the undeformed configuration. In addition, current electromechanics codes are often the result of *ad hoc* coupling methods between the electrophysiology and finite elasticity codes, limiting time integration to only first order numerical schemes and poor stability, although some approaches are known to address these stability issues (Niederer and Smith, 2008; Pathmanathan and Whiteley, 2009). This problem is common in other fields that use multiphysics (Keyes et al., 2013).

Our contributions in this paper are the following. First, we give a consistent derivation of the continuum equations of coupled electromechanics of the heart based on basic principles from geometry and physics and the clarification of the constitutive equations used. From this we show that there are no convective terms in the undeformed configuration and that the variety of deformation-dependent conduction tensors from literature are all special cases of a more general form that we present here. Second, we generalize Euler-based implicit-explicit schemes for electromechanics to higher order implicit-explicit Runge-Kutta schemes, based on the knowledge of fast/slow dynamics. Third, we explain on how to solve the resulting non-linear implicit equations from a general multiphysics perspective.

This paper is structured as follows. In section 2 we introduce the necessary notations and concepts and present the strong and weak form for the continuum electromechanics equations, followed by a brief discussion on how to discretize the weak form equations using finite elements in section 3. Next, we discuss on how to discretize the electromechanics equations in time using implicit-explicit schemes and how to solve the resulting non-linear equations in 4. Finally, in section 5 we explain how we implemented this using PETSc (Balay et al., 1997, 2016a,b) in our GEMS (Ghent ElectroMechanics Solver) code, and give examples of numerical results in section 6.

## 2. Physics

In this section we introduce the mathematical basis for physical modeling in the moving domain, distinguishing between the Eulerian and Lagrangian viewpoints. Then we show how the balance equations (i.e., physical conservation laws) need to be closed by constitutive equations. By imposing symmetry (e.g., a locally uniaxial medium), the constitutive equations involving tensors cannot be chosen freely, but need to be of certain form which we here propose and discuss. We conclude by splitting the equations in fast and slow components, which will be respectively treated implicitly and explicitly during time stepping in section 4. At the end of this section, we will have cast the modeling equations in variational form, suitable for use in the finite element approach.

### 2.1. Definitions and notation for geometrical concepts

To formulate the problem of electromechanics, it is important to understand the underlying geometry. Since we will consider continuum equations here, it is natural to consider them on a manifold, i.e., a “curved” space which locally resembles Euclidean space. For additional background material we refer to Marsden and Hughes (1994) and Frankel (2012).

Let B be the *material manifold* of dimension *m*. This is a reference manifold for our body. For an excitable surface, *m* = 2 and for a three-dimensional tissue, *m* = 3. On every patch of B, we define material coordinates *X*^*I*^, *I* = 1, .., *m*.

The space in which the body moves is given by the *spatial manifold* S (which is sometimes called the ambient or target manifold), of dimension *n*. For example, if an excitable surface is restricted to move in a plane, *n* = 2. However, in the general case where the tissue can move in 3D, *n* = 3. On every patch of S, we define spatial coordinates *x*^*i*^.

We will assume that we have a metric for these manifolds, which we denote by resp. *G* and *g*, so that we have Riemannian manifolds. In the simplest case (which we will use further) S will be n-dimensional Euclidean space, such that *x*^*i*^ are Cartesian coordinates *x, y, z*, and B will be an open subset of Euclidean m-dimensional space. However, a non-Euclidean metric on B can be important in growth and remodeling phenomena (Ozakin and Yavari, 2010), e.g., hypertrophy and thermoelasticity (Yavari, 2010).

A *configuration* of B is a mapping ϕ:B→S which represents the deformation of the body and we will often use the notation *x*^*i*^ = ϕ^*i*^. The set of all configurations of B is called the *configuration space* C and is an infinite-dimensional manifold.

The tangent map Tϕ:TB→TS,Tϕ(X,V) = (ϕ(*X*), *Dϕ*(*V*)) is called the *deformation gradient* *F* and is $F_{I}^{i}=\frac{\partial \phi^{i}}{\partial X^{I}}$ in components. This tells us how a tangent vector at a point X∈B transforms under ϕ.

Another important concept is the *deformation tensor* *C*, which is the pullback of the metric g: *C* = ϕ^*^g, or in components $C_{IJ}=F_{I}^{i}g_{ij}F_{J}^{j}$. Note that the squared infinitesimal distance between nearby points with coordinates *X*^*I*^ and *X*^*I*^+*dX*^*I*^ or *x*^*i*^ and *x*^*i*^+*dx*^*i*^ is $ds^{2}=g_{ij}dx^{i}dx^{j}=C_{IJ}dX^{I}dX^{J}$, showing that *C*_*IJ*_ is a measure for how length and angles between fixed pairs of points in the tissue change under a deformation. If we pull back the volume form *dv* on S to B, we get ϕ^*^dv = *JdV*, where *dV* is the volume form on B and $J=\frac{\sqrt{\det g}}{\sqrt{\det G}}\det F$ the Jacobian of the deformation.

The strain in the tissue will depend on how the current length and angles relate to the reference case, which is quantified by the *strain tensor* $E=\frac{\phi^{*}g-G}{2}$. Since ϕ is an isometry only if ϕ^*^g = *G*, *E* measures the deviation between the current deformation and an isometry.

In cardiac contraction, the configuration (or deformation) ϕ is time-dependent, which can be represented by a curve C in configuration space, i.e., a mapping $R\to C;t\to \phi_{t}$, called the *motion*. The *material velocity* and *acceleration* are then defined to be respectively the first and second time derivatives of the motion. Their components are given by $V^{i}=\frac{\partial \phi^{i}}{\partial t}$ and $A^{i}=\frac{\partial V^{i}}{\partial t}+\left(\gamma_{jk}^{i}{}^{\circ}\phi \right)V^{j}V^{k}$, where $\gamma_{jk}^{i}$ are connection coefficients on S. Since we use Euclidean space for S, we have $\gamma_{jk}^{i}=0$.

At this point, it is useful to discuss the Eulerian and Lagrangian viewpoints. Given the above definitions, any objects that are defined on B are called Lagrangian or material, while the concepts defined on S are called Eulerian or spatial. The Lagrangian and Eulerian point of view are equivalent, because anything that is defined in one can be transformed to the other. For cardiac tissue it is natural to use the Lagrangian framework. This has the advantage that we do not need convective derivatives in the description.

To model the cardiac microstructure, i.e., the fiber, sheet and normal direction, we will use *frame fields*, which are also called *vielbeins* in physics. Frame fields are a set of orthonormal vector fields. They span at each point of a manifold a basis for the tangent space. If *G* is the metric of our (material) manifold and ${\left\{E_{A}\right\}}_{A=1}^{m}$ the frame field, the orthonormality condition is

(1) $$\begin{array}{r} G\left(E_{A},E_{B}\right)=\delta_{AB}. \end{array}$$

The dual of the frame field is denoted *E*^*A*^ (with upper indices) and called the coframe field. It is defined to obey $E^{A}\left(E_{B}\right)=\delta_{B}^{A}$, such that it can be used to write the metric in the simple form

(2) $$\begin{array}{r} G=\overset{m}{\underset{A=1}{\sum }}E^{A}⊗E^{A}. \end{array}$$

We will denote the components of the frame field *E*_*A*_ in the coordinate basis by $E_{A}^{I}$ and of the coframe field *E*^*A*^ by $E_{I}^{A}$.

### 2.2. Balance equations

Although the bidomain and elasticity equations are well-known, we will still derive for consistency the equations of cardiac electromechanics here starting from basic continuum balance laws. This will allow us explicitly mention assumptions and approximations made, and to emphasize that cardiac electromechanics is more than just the sum of bidomain and elasticity equations, giving rise to more complicated constitutive equations (such as deformation-dependent conduction tensors).

Our starting point are physical conservation laws: balance of charge in the intra- and extracellular domains, no accumulation of total charge, balance of momentum, and the dynamics of the internal variables (such as gating variables and ionic concentrations):

(3a) $$\begin{array}{rc} \frac{\partial Q_{i}}{\partial t}+\text{DIV}J_{i} & =-I_{ion}, \end{array}$$

(3b) $$\begin{array}{rc} \frac{\partial Q_{e}}{\partial t}+\text{DIV}J_{e} & =I_{ion}, \end{array}$$

(3c) $$\begin{array}{rc} \frac{\partial \left(Q_{i}+Q_{e}\right)}{\partial t} & =0, \end{array}$$

(3d) $$\begin{array}{rc} \rho_{\text{Ref}}A-\text{DIV}P-\rho_{\text{Ref}}B & =0, \end{array}$$

(3e) $$\begin{array}{rc} \frac{\partial \Gamma }{\partial t} & =R, \end{array}$$

where *Q*_*i*_ and *Q*_*e*_ are the intra- and extracellular charge densities, *J*_*i*_ and *J*_*e*_ are the intra- and extracellular current densities, ρ_Ref_ is the reference mass density, *A* is the acceleration, *P* the first Piola-Kirchhoff stress tensor, *B* is the body force (e.g., gravity), Γ is a column matrix of the internal variables and *R* are their reaction rates. Note that all quantities live on the material manifold B and DIV is the divergence operator on B.

The assumptions in the bidomain formulation are the following. First, the cell membrane can be modeled as a capacitor: *Q*_*i*_−*Q*_*e*_ = 2*C*_*m*_*V*_*m*_, where *C*_*m*_ is the capacitance per volume and *V*_*m*_ = *V*_*i*_−*V*_*e*_ the transmembrane voltage. Second, the intra- and extracellular space are ohmic conductors, with intra- and extracellular conductivities Σ_*i*_ and Σ_*e*_ . Thus we get the following set of equations:

(4a) $$\begin{array}{r} \frac{\partial \left(C_{m}V_{m}\right)}{\partial t}+\text{DIV}\left(\Sigma_{i}\cdot \text{GRAD}V_{m}\right)+\text{DIV}\left(\Sigma_{i}\cdot \text{GRAD}V_{e}\right)=-I_{ion}, \end{array}$$

(4b) $$\begin{array}{r} \text{DIV}\left(\Sigma_{i}\cdot \text{GRAD}V_{m}\right)+\text{DIV}\left(\left(\Sigma_{i+e}\right)\cdot \text{GRAD}V_{e}\right)=0, \end{array}$$

(4c) $$\begin{array}{r} \frac{\partial \Gamma }{\partial t}=R, \end{array}$$

(4d) $$\begin{array}{r} \rho_{\text{Ref}}A-\text{DIV}P-\rho_{\text{Ref}}B=0. \end{array}$$

An assumption often made in cardiac mechanics is the neglect of the inertial term ρ_Ref_*A*. This is justified because sound waves occur on a much faster time scale than the electrical waves in cardiac tissue: the ratio of the speed of sound to conduction velocity is around 25. This was also validated numerically in an electromechanical model of a 1D fiber (Whiteley et al., 2007).

### 2.3. Constitutive equations

To close Equations (4) we need to specify constitutive equations for Σ_*i*_, Σ_*e*_, *I*_*ion*_, *R*, and *P*. We will only consider the dependencies as pointwise functions of material position *X*, transmembrane potential *V*_*m*_, internal variables Γ and deformation *C*:

(5a) $$\begin{array}{r} \Sigma_{i}={\hat{\Sigma }}_{i}\left(X,C\right), \end{array}$$

(5b) $$\begin{array}{r} \Sigma_{e}={\hat{\Sigma }}_{e}\left(X,C\right), \end{array}$$

(5c) $$\begin{array}{r} I_{ion}={Î}_{ion}\left(X,V_{m},\Gamma ,C\right), \end{array}$$

(5d) $$\begin{array}{r} R=\hat{R}\left(X,V_{m},C\right) \end{array}$$

(5e) $$\begin{array}{r} P=FŜ\left(X,\Gamma ,C\right). \end{array}$$

Instead of working with a function $\hat{P}$ for the first Piola-Krichhoff stress tensor, we directly work with a function Ŝ for the second Piola-Kirchhoff stress tensor, because it is symmetric. It is also possible that the material capacitance depends on deformation, and therefore we write *C*_*m*_ = Ĉ_*m*_(*X, C*). Based on the symmetries of the material we can deduce more specific representations for the scalar (Î_*ion*_, $\hat{R}$, and Ĉ_*m*_) and symmetric second order tensor functions (${\hat{\Sigma }}_{i}$, ${\hat{\Sigma }}_{e}$, Ŝ). Because of the specific microstructure of cardiac tissue, we only focus on orthotropic materials, but more general symmetries based on crystal groups are possible (Smith, 2012). For the following we will use the notation {_*E*_*A*_}*A*∈{*F, S, N*}_ for the local fiber, sheet and sheet normal directions.

Let us start with the scalar functions. It can be shown (Itskov, 2013) that every scalar-valued function of a symmetric rank-2 tensor *M*, such as the deformation tensor *C*, the second Piola-Kirchhoff stress tensor *S* and the conduction tensors Σ_*i*_, Σ_*e*_, which is invariant under orthotropic symmetries can be written as a function of the seven invariants $\left\{M_{FF},M_{SS},M_{NN},{\left(M_{FS}\right)}^{2},{\left(M_{FN}\right)}^{2},{\left(M_{SN}\right)}^{2},M_{FS}M_{SN}M_{NF}\right\}$. If det(*M*) = 1, (e.g., when *M* is the deformation tensor of an incompressible material), these seven invariants are not independent anymore and we can leave out the last one. In that case our scalar constitutive equations would be a function of the six invariants $\left\{M_{FF},M_{SS},M_{NN},{\left(M_{FS}\right)}^{2},{\left(M_{FN}\right)}^{2},{\left(M_{SN}\right)}^{2}\right\}$. Often Î_*ion*_ and $\hat{R}$ are taken to be a function of the fiber stretch $\lambda =\sqrt{C_{FF}}$ only, see for example Niederer et al. (2006) and Panfilov et al. (2007).

Orthotropic tensor-valued functions *T* of a symmetric tensor *M* can be shown to be of the form (Itskov, 2013)

(6) $$\begin{array}{l} \hat{T}(M)=\sum_{A\in \{F,S,N\}}[{\hat{\alpha }}_{A}\left(E_{A}⊗E_{A}\right)\\ \;+\frac{{\hat{\beta }}_{A}}{2}\left(M\cdot E_{A}⊗E_{A}+E_{A}⊗E_{A}\cdot M\right)\\ \;+\frac{{\hat{\gamma }}_{A}}{2}\left(M^{2}\cdot E_{A}⊗E_{A}+E_{A}⊗E_{A}\cdot M^{2}\right)\\ \;+\frac{{\hat{\delta }}_{A}}{2}\left(M\cdot E_{A}⊗E_{A}-E_{A}⊗E_{A}\cdot M\right)\\ \;+\frac{{\hat{\epsilon }}_{A}}{2}\left(M^{2}\cdot E_{A}⊗E_{A}-E_{A}⊗E_{A}\cdot M^{2}\right)], \end{array}$$

where $\hat{\alpha }$, $\hat{\beta }$, $\hat{\gamma }$, $\hat{\delta }$, and $\hat{\epsilon }$ are now scalar-valued functions of *M*. Note that for $\hat{T}\left(M\right)$ symmetric ${\hat{\delta }}_{A}={\hat{\epsilon }}_{A}=0$ while for $\hat{T}\left(M\right)$ antisymmetric ${\hat{\alpha }}_{A}={\hat{\beta }}_{A}={\hat{\gamma }}_{A}=0$.

When we write out this expression in components of the *E*_*A*_ frame (*A*, *B* ∈{*F, S, N*}, no summation implied) we get:

(7) $$\begin{array}{r} {\hat{T}}_{AB}\left(M\right)=\frac{{\hat{\alpha }}_{A}+{\hat{\alpha }}_{B}}{2}\delta_{AB}+\frac{{\hat{\beta }}_{A}+{\hat{\beta }}_{B}+{\hat{\delta }}_{A}-{\hat{\delta }}_{B}}{2}M_{AB}\\ \;+\frac{{\hat{\gamma }}_{A}+{\hat{\gamma }}_{B}+{\hat{\epsilon }}_{A}-{\hat{\epsilon }}_{B}}{2}{\left(M^{2}\right)}_{AB}. \end{array}$$

The second Piola-Kirchhoff stress tensor *S* is symmetric and in the case that it is hyperelastic (such that ${Ŝ}^{IJ}\left(C\right)=2\frac{\partial \hat{\psi }}{\partial C_{IJ}}$, where $\hat{\psi }$ is a function of the invariants), the constitutive equation simplifies to

(8) $$\begin{array}{l} \hat{S}(C)=\sum_{A\in \{F,S,N\}}[{\hat{\alpha }}_{A}\left(E_{A}⊗E_{A}\right)+\frac{{\hat{\beta }}_{A}}{2}(C\cdot E_{A}⊗E_{A}\\ \;+E_{A}⊗E_{A}\cdot C)]+\hat{\gamma }C^{2}. \end{array}$$

For ventricular cardiac tissue, the Guccione (Guccione et al., 1995) and Holzapfel-Ogden (Holzapfel and Ogden, 2009) constitutive equations are popular choices.

Throughout the literature on cardiac electromechanical modeling, several deformation-dependent conduction tensors have been proposed. The simplest form is obtained by making the conduction coefficients Σ_*A*_ dependent on the stretch along the principal material directions: with $\lambda_{A}=\sqrt{C\left(E_{A},E_{A}\right)}$,

(9) $$\begin{array}{r} \hat{\Sigma }\left(C\right)=\underset{A\in \left\{F,S,N\right\}}{\sum }{\hat{\Sigma }}_{A}\left(\lambda_{A}\right)E_{A}⊗E_{A} \end{array}$$

Examples for these are ${\hat{\Sigma }}_{A}\left(\lambda_{A}\right)=\Sigma_{A}$, i.e., deformation-independent or “gap-junction based” conduction(Bakir and Dokos, 2015) or ${\hat{\Sigma }}_{A}\left(\lambda_{A}\right)=\frac{\Sigma_{A}}{\lambda_{A}^{2}}$ (Colli Franzone et al., 2016). Yet another form for the conduction tensor can be found in Bakir and Dokos (2015), which they call “spatially based” conduction:

(10) $$\begin{array}{r} \hat{\Sigma }\left(C\right)=JU^{-1}\cdot \left(\underset{A\in \left\{F,S,N\right\}}{\sum }\Sigma_{A}\left(\lambda_{A}\right)E_{A}⊗E_{A}\right)\cdot U^{-T}, \end{array}$$

where *U* is the right stretch tensor, i.e., $U=\sqrt{C}$. A related form is (Sachse, 2004):

(11) $$\begin{array}{r} \hat{\Sigma }\left(C\right)=W\cdot \left(\underset{A\in \left\{F,S,N\right\}}{\sum }\Sigma_{A}\left(\lambda_{A}\right)E_{A}⊗E_{A}\cdot \right)W^{T}, \end{array}$$

where *W* = *U*^−1^(1+θ(*U*−1)) and θ∈[0, 1] is a parameter which reduces this conduction tensor to the “spatial based” conduction for θ = 0 (apart from the Jacobian factor) and to a “gap-junction based” conduction for θ = 0.

In Göktepe and Kuhl (2010) and Göktepe et al. (2013) the following transversely isotropic form

(12) $$\begin{array}{r} \hat{\Sigma }\left(C\right)=\Sigma_{iso}C^{-1}+\Sigma_{ani}E_{F}⊗E_{F} \end{array}$$

was used and in Plank et al. (2013):

(13) $$\begin{array}{r} \hat{\Sigma }\left(C\right)=\left(\underset{A\in \left\{F,S,N\right\}}{\sum }\Sigma_{A}E_{A}⊗E^{A}\right)\cdot C^{-1}. \end{array}$$

This variety of deformation-dependent conduction tensors is mostly a consequence of the assumptions that were made about the conduction coefficients, for example one assumes that the conduction coefficients are constant in the spatial or in the material frame. However, nothing says a priori if these should even be constant. So to have realistic deformation-dependent conduction tensors relationships, the conduction coefficients should be based on measurements with different deformations.

### 2.4. Variational formulation

In view of the time-integration methods which will be presented in section 4.1, let us split *R* in fast processes (to be treated implicitly) and slow processes: *R* = *R*_*I*_+*R*_*E*_. Furthermore, let *P*_*appl*_ denote the applied pressure on the pressure boundary of the deformation ϕ (e.g., the fluid pressure at the endocardial surfaces). Writing the fast processes on the left-hand side and the slow processes at the right-hand side, the weak or variational form for electromechanics can be written as: find *V*_*m*_, *V*_*e*_, Γ, ϕ such that

(14a) $$\begin{array}{r} \int_{B}\delta V_{m}\frac{\partial V_{m}}{\partial t}dV+\int_{B}\delta V_{m}{|}_{I}{\left(\Sigma_{i}\right)}^{IJ}\left(V_{m}{|}_{J}+V_{e}{|}_{J}\right)dV=-\int_{B}\delta V_{m}I_{ion}dV \end{array}$$

(14b) $$\begin{array}{r} \int_{B}\delta V_{e}{|}_{I}\left({\left(\Sigma_{i}\right)}^{IJ}V_{m}{|}_{J}+{\left(\Sigma_{i+e}\right)}^{IJ}V_{e}{|}_{J}\right)dV=0 \end{array}$$

(14c) $$\begin{array}{r} \int_{B}\delta \Gamma \left(\frac{\partial \Gamma }{\partial t}-R_{I}\right)dV=\int_{B}\delta \Gamma R_{E}dV \end{array}$$

(14d) ∫B δϕi|IPiIdV+∫∂NB δϕiPapplJ(F−1)INiIdS=0,

for all test functions δ*V*_*m*_, δ*V*_*e*_, δΓ, δϕ. The notation |_*I*_ was introduced for the *I*'th component of the covariant derivative, i.e., $\delta V_{e}{|}_{I}=\frac{\partial V_{m}}{\partial X^{I}}$ and $\delta \phi^{i}{|}_{I}=\frac{\partial \left(\delta \phi^{i}\right)}{\partial X^{I}}+\gamma_{jk}^{i}\delta \phi^{j}F_{I}^{k}$ (again, for Euclidean S the connection γ vanishes).

Note that we can write any left-hand side of (14) in the following form:

(15) $$\begin{array}{r} \int_{B}\left(v\cdot f_{0}+\nabla v:f_{1}\right)dV+\int_{\partial_{N}B}v\cdot g_{0}dS \end{array}$$

where *v* represents any of the test functions and *f*_0_, *f*_1_, and *g*_0_ are general functions of *V*_*m*_, *V*_*e*_, Γ, and ϕ, their gradients and time derivatives, time and spatial coordinates. More specifically, we can summarize all the fast physics by pointwise functions in the following table:

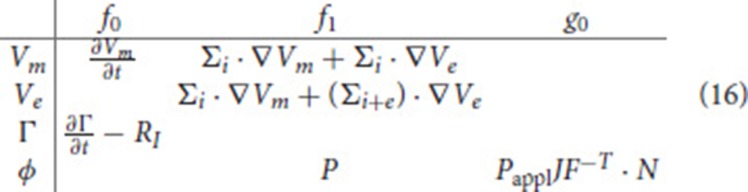



For implicit time integration we will also need the Jacobian of the left-hand side. Its action on the increments Δ*V*_*m*_, Δ*V*_*e*_, ΔΓ, and Δϕ is given by

(17a) $$\begin{array}{r} \int_{B}\delta V_{m}\gamma \Delta V_{m}dV+\int_{B}\delta V_{m}{|}_{I}{\left(\Sigma_{i}\right)}^{IJ}\Delta V_{m}{|}_{J}dV \end{array}$$

(17b) $$\begin{array}{r} \int_{B}\delta V_{m}{|}_{I}{\left(\Sigma_{i}\right)}^{IJ}\Delta V_{e}{|}_{J}dV \end{array}$$

(17c) $$\begin{array}{r} \int_{B}\delta V_{e}{|}_{I}{\left(\Sigma_{i}\right)}^{IJ}\Delta V_{m}{|}_{J}dV \end{array}$$

(17d) $$\begin{array}{r} \int_{B}\delta V_{e}{|}_{I}{\left(\Sigma_{i+e}\right)}^{IJ}\Delta V_{e}{|}_{J}dV \end{array}$$

(17e) $$\begin{array}{r} \int_{B}\delta \Gamma \left(\gamma -\frac{\partial R_{I}}{\partial \Gamma }\right)\Delta \Gamma dV \end{array}$$

(17f) $$\int_{B}\delta {\phi^{i}|}_{I}A_{i\;j}^{I\;J}{\Delta \phi^{j}|}_{J}dV+\int_{\partial_{N}B}\delta \phi^{i}P_{\text{appl}}B_{ij}^{J}{\Delta \phi^{j}|}_{J}dS$$

where γ is the shift factor determined by the numerical integration scheme (for example, for backward Euler with time step *h*, γ = *h*^−1^) and

(18) $$\begin{array}{l} B_{ij}^{J}=\frac{\partial \left(J{\left(F^{-1}\right)}^{I}{}_{i}N_{I}\right)}{\partial F^{j}{}_{J}}=JN_{I}{\left(F^{-1}\right)}^{I}{}_{i}{\left(F^{-1}\right)}^{J}{}_{j}\\ \;-{\left(F^{-1}\right)}^{I}{}_{j}{\left(F^{-1}\right)}^{J}{}_{i}, \end{array}$$

and

(19) $$A_{i\;j}^{I\;J}=\frac{\partial P_{i}^{I}}{\partial F^{j}{}_{J}}$$

is called the first elasticity tensor (Marsden and Hughes, 1994).

The expressions (17) can generally be written as

(20) $$\begin{array}{l} \int_{B}\left[\begin{array}{cc} v^{T} & \nabla v^{T} \end{array}\right]\left[\begin{array}{cc} f_{0,0} & f_{0,1}\\ f_{1,0} & f_{1,1} \end{array}\right]\left[\begin{array}{c} w\\ \nabla w \end{array}\right]dV+\int_{\partial_{N}B}\left[v^{T}\right]\left[\begin{array}{cc} g_{0,0} & g_{0,1} \end{array}\right]\left[\begin{array}{c} w\\ \nabla w \end{array}\right]dS \end{array}$$

and the pointwise Jacobians can be summarized as

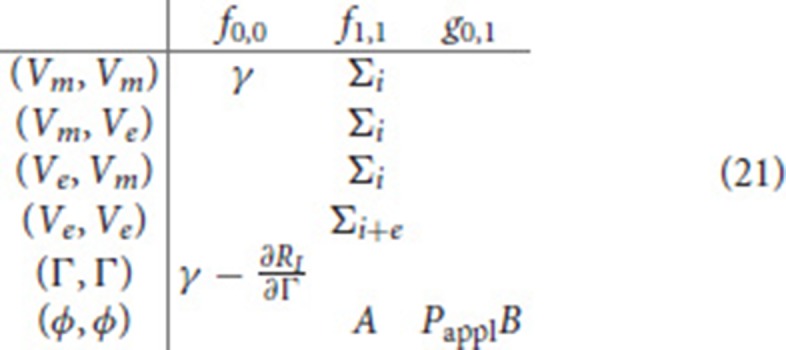



where for example (*V*_*m*_, *V*_*e*_) refers to the derivative of the weak equation for *V*_*m*_ w.r.t. *V*_*e*_.

## 3. Discretization

In this section we apply standard methods to express the variational equations in a finite element basis, to obtain a large non-linear system to solve instead of continuum partial differential equations.

We will use the finite element method (Ciarlet, 2002; Brenner and Scott, 2007; Zienkiewicz et al., 2013) to spatially discretize the weak forms (14). Let the manifold B be triangulated into *E m*-simplices ${\left\{K^{e}\right\}}_{e=1}^{E}$ (cells/elements), each diffeomorphic to the standard *m*-simplex $\hat{K}$ (with coordinates ξ^Î^): for each *e* there is a coordinate map ${\hat{X}}^{e}:\hat{K}\to K^{e}$ for which their element Jacobians ${\left(J^{e}\right)}_{Î}^{I}=\frac{\partial {\hat{X}}^{I}}{\partial \xi^{Î}}$ and their inverse exist (and are continuous). If we also choose a function space *P* and a basis for the dual space Σ over each element, the triple (*K, P*, Σ) defines the finite element (Ciarlet, 2002). Here we will only use 1^*st*^ order Lagrange elements (Brenner and Scott, 2007). Let ${\left\{\varphi_{p}\right\}}_{p=1}^{\dim P}$ denote the basis functions for *P* and let ${\left\{\xi_{q}\right\}}_{q=1}^{Q}$ and ${\left\{w_{q}\right\}}_{q=1}^{Q}$ be the quadrature points of a quadrature rule with *Q* quadrature points (e.g., Gauss-Jacobi in the case of simplices Karniadakis and Sherwin, 2013). Then we can define the element basis evaluation, derivative and integration matrices as ${\left(B^{e}\right)}_{qp}=\varphi_{p}\left(\xi_{q}\right)$, ${\left(D_{I}^{e}\right)}_{qp}=\frac{\partial \varphi_{p}}{\partial \xi^{Î}}\left(\xi_{q}\right){\left({\left(J^{e}\right)}^{-1}\right)}_{I}^{Î}$ and ${\left(W^{e}\right)}_{qp}=\delta_{qp}w_{q}\det\left(J_{e}\right)$

Following (Brown, 2010; Knepley et al., 2013) we discretize the volume terms

(22) $$\begin{array}{r} \int_{B}\left(v\cdot f_{0}+\nabla v:f_{1}\right)dV \end{array}$$

as

(23) $$\begin{array}{r} \underset{e}{\sum }E_{e}^{T}\left[{\left(B^{e}\right)}^{T}W^{e}\Lambda^{e}\left(f_{0}\right)+\sum_{I}{\left(D_{I}^{e}\right)}^{T}W^{e}\Lambda^{e}\left(f_{1}^{I}\right)\right], \end{array}$$

where $E_{e}$ is the element restriction operator and Λ^*e*^ transforms a function into function evaluations at the quadrature points. Note that evaluation of a field *u* at the quadrature points are evaluated as $u^{e}=B^{e}E_{e}u$ and their derivatives as $\nabla_{I}u^{e}=D_{I}^{e}E_{e}u$.

The boundary integrals

(24) $$\begin{array}{r} \int_{\partial_{N}B}v\cdot g_{0}dS \end{array}$$

are discretized as

(25) $$\begin{array}{r} \underset{f}{\sum }E_{e\left(f\right)}^{T}\left[{\left(B^{e\left(f\right)}\right)}^{T}W^{f}\Lambda^{e\left(f\right)}\left(g_{0}\right)\right], \end{array}$$

where *e*(*f*) refers to the neighboring element of *f*, i.e., we evaluate at the quadrature points of the face using the neighboring element's basis functions and field coefficients.

## 4. Algorithms

In this section we present IMEX integration schemes, the resulting non-linear equations and approaches to solve them numerically for the specific structure of the electromechanical equations.

### 4.1. Time integration using IMEX schemes

For systems that have multiple time scales that are well-separated, we have to choose a time scale that we are interested in. In studying the long term or slowly varying behavior, the fast transient processes don't need to be fully resolved, as these decay rapidly. These systems are called stiff (see Söderlind et al., 2015 for a discussion on stiffness). Note that in discretized PDEs, the fastest time scale often comes in the form of a Courant-Friedrichs-Levy limit (Courant et al., 1928), making it mesh-dependent.

Explicit schemes require the time step to be of the same order as the fastest process for stability, so they are very inefficient for stiff systems. Implicit schemes can step over those fast processes, but the downside is that they produce large fully coupled non-linear systems. Implicit-Explicit (IMEX) schemes combine the best of both worlds: they integrate the fast processes implicitly and the slow processes explicitly. A class of IMEX methods are Additive Runge-Kutta Implicit-Explicit (ARKIMEX) schemes (Ascher et al., 1997; Kennedy and Carpenter, 2001; Giraldo et al., 2013). They combine two s-stage methods (ERK and (ES)DIRK), summarized by two Butcher tableaus (Butcher, 2016)

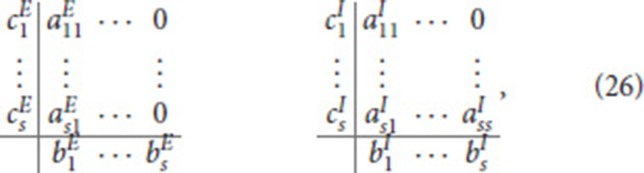



additively to integrate equations of the following form

(27) $$\begin{array}{r} Mẏ=f^{I}\left(y,t\right)+f^{E}\left(y,t\right), \end{array}$$

where *y*:*I*→*R*^*N*^ describes the evolution of the discretized state, *f*^*I*^ and *f*^*E*^ are resp. the implicitly and the explicitly treated functions and *M* is a mass matrix. The implicit function contains the fast or stiff physics, whereas the explicit function contains the slow or non-stiff physics. Often *f*^*I*^ is linear and *fE* non-linear. The i-th stage value *Y*_*i*_ can then be computed as

(28) $$\begin{array}{r} Y_{i}=y_{n}+h\overset{i-1}{\underset{j=1}{\sum }}a_{ij}^{E}{Ẏ}_{j}^{E}+h\overset{i}{\underset{j=1}{\sum }}a_{ij}^{I}{Ẏ}_{j}^{I}, \end{array}$$

where the implicit and explicit stage derivates are given by ${Ẏ}_{i}^{I}=M^{-1}f^{I}\left(Y_{i},t_{n}+c_{i}h\right)$ and ${Ẏ}_{i}^{E}=M^{-1}f^{E}\left(Y_{i},t_{n}+c_{i}h\right)$. The difference between both terms is that the stage *Y*_*i*_ depends on only previous stages for the explicit part, but also on the current stage for the implicit part. The numerical constants $a_{ij}^{I}$, $a_{ij}^{E}$ follow from the chosen integration scheme, see the Butcher tableaus (26).

After rearranging, Equation (28) produces a non-linear equation in *Y*_*i*_, if the $a_{ii}^{I}\neq 0$:

(29) $$\begin{array}{r} M\gamma \left(Y_{i}-Z_{i}\right)-f^{I}\left(Y_{i},t_{n}+c_{i}h\right)=0, \end{array}$$

where γ is the shift factor determined by the numerical integration scheme (for example, for backward Euler with time step *h*, γ = *h*^−1^). The Jacobian for this equation is

(30) $$\begin{array}{r} \gamma M-\frac{\partial f^{I}}{\partial y}\left(Y_{i},t_{n}+c_{i}h\right) \end{array}$$

and is used while iteratively solving Equation (29) for *Y*_*i*_. Thereafter, the implicit stage derivative can be simply found as

(31) $$\begin{array}{r} {Ẏ}_{i}^{I}=\gamma \left(Y_{i}-Z_{i}\right) \end{array}$$

and the explicit stage derivative by evaluating the explicit function

(32) $$\begin{array}{r} {Ẏ}_{i}^{E}=M^{-1}f^{E}\left(Y_{i},t_{n}+c_{i}h\right). \end{array}$$

The solution at the next time step is then calculated as

(33) $$\begin{array}{r} y_{n+1}=y_{n}+h\overset{s}{\underset{i=1}{\sum }}b_{i}^{E}{Ẏ}_{i}^{E}+h\overset{s}{\underset{j=1}{\sum }}b_{i}^{I}{Ẏ}_{i}^{I}. \end{array}$$

Note that if *f*^*I*^ = 0 we have a purely explicit scheme and if *f*^*E*^ = 0 we have a purely implicit scheme. In order to avoid the need to invert *M*, we will only use schemes for which $a_{si}^{I}=b_{i}^{I}$ and $a_{si}^{E}=b_{i}^{E}$, the so-called globally stiffly accurate schemes (Boscarino et al., 2013). Then, the completion step (33) can be skipped. For a more thorough discussion on the technical aspects, we refer to Kennedy and Carpenter (2001). In the context of electrophysiology they were previously applied only to single cell models, where they have been shown to outperform other integration schemes (Spiteri and Dean, 2008).

### 4.2. Non-linear solvers

The IMEX schemes allow us to put some of the complicated non-linear dependencies in the right-hand sides, making the implicit solve easier. If we make the following assumptions, we can essentially solve the whole non-linear system by solving each subproblem one after another: the ionic current, the stretch-dependent terms in the cell models and dependence of the tension variables on *Ca*_*i*_ or *V*_*m*_ must be in the RHS. Now we can solve for the stage values by doing the following: first solve the active tension internal variable equations, then solve the mechanical equations (14d), then solve the bidomain equations ((14a) and (14b)) together and finally solve the electrophysiological internal variable equations (14c). This approach is nothing more than the non-linear Gauss-Seidel method applied to the fields:

**Algorithm 1:** Nonlinear Gauss-Seidel

Given initial $u={\left(u_{1},\cdots \;,u_{n}\right)}^{T}$
**for** *k* = 1, ⋯, *n* **do**
Solve $F_{k}\left(u_{1}^{*},\cdots \;,u_{k}^{*},\cdots \;,u_{n}\right)=0$ for $u_{k}^{*}$
**end for**

During this process, we solve the bidomain and, if possible, the implicit internal variables equations with a linear solver (to be specified below), while we solve the non-linear mechanical equations with Newton's method. If for some reason some of the above assumptions do not hold and coupling between variables is strong enough, more Gauss-Seidel sweeps are done to converge. Alternatively, one could use the above algorithm as a non-linear preconditioner (Liu and Keyes, 2015).

### 4.3. Linear solvers and preconditioners

#### 4.3.1. Bidomain

We solve the discretized bidomain equations with conjugate gradients preconditioned by block preconditioners (Sundnes et al., 2002; Pennacchio and Simoncini, 2009; Bernabeu et al., 2010; Pavarino and Scacchi, 2011). For this we use PETSc's FieldSplit preconditioner, allowing us to flexibly choose between different strategies (Brown et al., 2012) from the command line. Both blocks are preconditioned with one V-cycle of PETSc's native algebraic multigrid preconditioner (GAMG). If no Dirichlet boundary conditions are given for the extracellular voltage, we also provide the constant nullspace vector to the respective block solve.

#### 4.3.2. Mechanics

We solve the linearized elasticity equations arising from Newton's method with conjugate gradients, preconditioned with PETSc's algebraic multigrid preconditioner. The difference here with previous work (Franzone et al., 2015; Gurev et al., 2015; Augustin et al., 2016) is that this algebraic multigrid preconditioner uses smoothed aggregation (Vaněk et al., 1996), which is more efficient for elasticity problems (Van et al., 2001; Adams, 2002). We provide the rigid body modes to PETSc's GAMG preconditioner to obtain more accurate coarse spaces, resulting in a significant drop in iterations. Here we use a full multigrid cycle as this also helps in lowering the number of iterations of the linear solver at the expense of only a small percentage more work than a single V-cycle.

#### 4.3.3. Internal variables

As the internal variables on different points are completely decoupled these can be solved easily as small linear systems. Very often these systems are even diagonal, for example when most of the stiffness comes from the gating variables.

## 5. Implementation: GEMS

### 5.1. Source code in C using PETSc

We implemented our code in C using the PETSc library (Balay et al., 1997, 2016a,b). This allows us to have a large choice of scalable and efficient algorithms and data structures for the solution of time-dependent PDE's, which can be easily changed or finetuned through command line options. By using PETSC's unstructured mesh data structure, we can easily read and write common mesh formats, (re)distribute meshes and associated data and we have access to powerful solvers which need access to mesh and field information (e.g., multigrid and block preconditioners). More specifically, we used DMPlex (Isaac and Knepley, 2015; Knepley et al., 2015; Lange et al., 2015) for mesh management and PetscFE for finite element technology, TS (Abhyankar, 2014) for time stepping, SNES for non-linear solvers and KSP/PC for linear solvers and preconditioners. Input and output routines are coupled to PETSc. Meshes can be read in through DMPlex if it is of the ExodusII (Schoof and Yarberry, 1994), Gmsh (Geuzaine and Remacle, 2009), CGNS (Poirier et al., 1998), MED (Open CASCADE, 2017), Fluent Case (Fluent, 2006), or PLY (Wikipedia, 2017) file format. Alternatively, meshes can also be created by giving the vertex numbers per cell and vertex coordinates. Output can be generated using the builtin PETSc viewers. For example, DM (mesh) and Vec (representing discrete fields) objects can be stored as HDF5 (The HDF Group, 1997-2017) data, which can be read by ParaView (Ayachit, 2015) or VisIt (Childs et al., 2012) with XDMF metadata (Kitware, 2017). The extensible nature of PETSc also makes it possible to implement new solvers and use them through PETSc. This way we implemented a SNES solver called SNESFieldSplit, which is the non-linear block Gauss-Seidel solver we discussed in 4.2. Once this solver knows about the field layout and the equations per field through the DM, it can automatically do the subsolves. This is the non-linear equivalent to PCFieldSplit (Brown et al., 2012), already in PETSc.

### 5.2. Main GEMS classes and usage

The most important part of our GEMS library is the *GEMSModel* class. It is responsible for providing all the model-dependent information such as pointwise residuals and Jacobians, discretizations, null spaces, and initial guess/conditions to the appropriate PETSc classes. Current subclasses include *GEMSModelMonodomain, GEMSModelBidomain, GEMSModelElasticity, GEMSModelElectromechanics* (combining monodomain and quasi-static elasticity), and *GEMSModelFibres* (to create rule-based fiber directions based on solving Laplace equations, following Bayer et al., 2012).

Typical usage for a non-linear problem is illustrated in 1. Note that nothing should be done extra to run simulations in different dimensions besides changing the mesh, which can be as simple as just changing the filename of the mesh. The …FromOptions(…) functions are meant to be configured from the command line or options file. For example, if the GEMSModel should be changed to GEMSModelMonodomain, the option *-gemsmodel_type monodomain* would be added to the command line or options file.

Listing 1 **Typical usage of the GEMSModel class**

 

    MPI_Comm  comm;

    SNES      snes;

    DM        dm;

    Vec       u;

    GEMSModel model;

  

    /^*^ Initialize GEMS, PETSc, MPI, read

       options ^*^/

    GEMSInitialize(&argc, &argv, NULL, help);

    comm = PETSC_COMM_WORLD;

  

    /^*^ Create a DMPlex using, e.g.,

    DMPlexCreateFromFile() ^*^/

    DMPlexCreate... (comm, ... , &dm);

  

    /^*^ Create and configure a GEMSModel ^*^/

    GEMSModelCreate(comm, &model);

    GEMSModelSetFromOptions(model);

    /^*^ Set model-specific discretizations and

    equations in the DM ^*^/

    GEMSModelSetUpDiscretization(model, dm);

    /^*^ Create model-specific near-null space

       (this is used by GAMG) ^*^/

    GEMSModelCreateNearNullSpace(model, dm,

       NULL);

  

    /^*^ Create and initialize the solution

       vector ^*^/

    DMCreateGlobalVector(dm, &u);

    PetscObjectSetName((PetscObject)u,

       ‘‘solution’’);

    ModelInitializeSolutionVector(model, dm,

       u);

  

    /^*^ Use DMPlex's internal FEM routines ^*^/

    DMSNESSetBoundaryLocal(dm,

      DMPlexSNESComputeBoundaryFEM, NULL);

    DMSNESSetFunctionLocal(dm,

      DMPlexSNESComputeResidualFEM, NULL);

    DMSNESSetJacobianLocal(dm,

      DMPlexSNESComputeJacobianFEM, NULL);

  

    /^*^ Create and configure the nonlinear

       solver and solve ^*^/

    SNESCreate(comm, &snes);

    SNESSetDM(snes, dm);

    SNESSetFromOptions(snes);

    SNESSolve(snes, NULL, u);

  

    /^*^ View the mesh ^*^/

    DMViewFromOptions(dm, NULL, ‘‘-dm_view’’);

  

    /^*^ View the solution ^*^/

    VecViewFromOptions(u, NULL, ‘‘-sol_vec_

    ␣␣view’’);

    /^*^ Clean up ^*^/

    SNESDestroy(&snes);

    VecDestroy(&u);

    ModelDestroy(&model);

    DMDestroy(&dm);

    GEMSFinalize();

 

Further we have a class for the electrophysiological 0D cell models called GEMSCellModel. Its only function is to give the pointwise implicit and explicit functions, Jacobian and initial conditions. Currently implemented cell models include FitzHugh-Nagumo (FitzHugh, 1961; Nagumo et al., 1962) and Ten Tusscher-Panfilov 2006 (ten Tusscher and Panfilov, 2006) models.

### 5.3. Comparison to other cardiac solvers

One of the main features of GEMS is, that it uses PETSc (and other third party packages it interfaces) as much as possible and not just as a linear algebra solver. In particular it uses the DM object prominently, which makes it easy to input/output meshes and field data in various formats, feed field and mesh data to various advanced (non)linear, often consisting of combinations of subsolvers, etc (for example, the block preconditioners for bidomain or incompressible elasticity in which each field has a different preconditioner and linear iterative solver). These solvers (and their subsolvers) can then be configured just from command line options, without recompiling. Thus it strives for maximal flexibility and easy experimentation. Other cardiac solvers such as Chaste (Mirams et al., 2013) or Continuity (Continuity, 2018) have already existed for many years and have functionalities such as reading generic cell models through CellML and solving mechanics. But the typical approach to electromechanics is first order operator splitting with separate codes for mechanics and electrophysiology. Our library was built with a flexible approach to coupling between different physics from the beginning. To specify a problem we start with a coupled set of equations (defined by pointwise residuals, right hand sides and Jacobians) and through command line options we can configure the solvers. This makes experimentation with different combinations of solvers a whole lot easier and also makes it possible to use higher order integration schemes.

## 6. Numerical results

### 6.1. Electrophysiology

**Figure 1:**
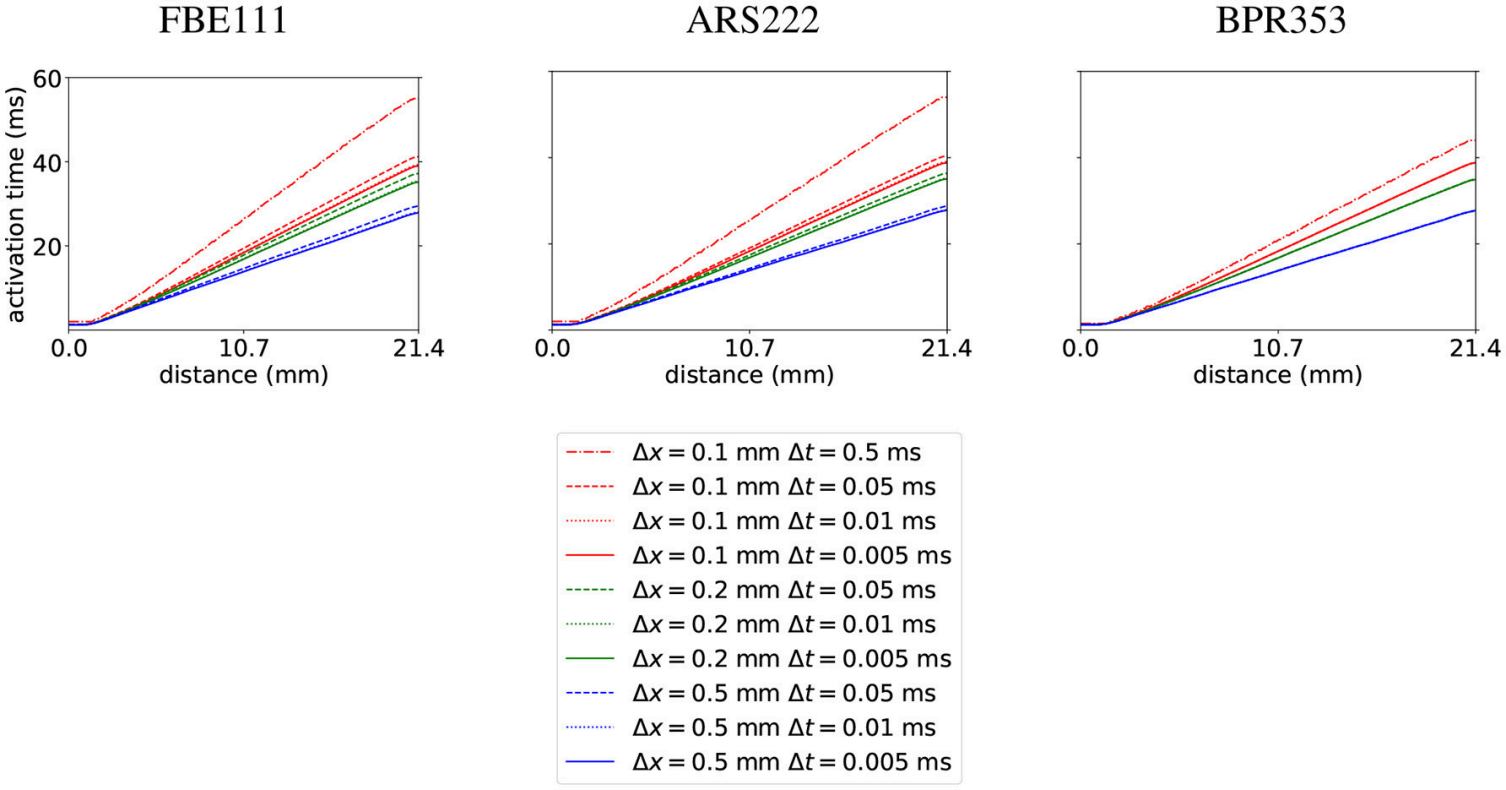
Activation times calculated with the FBE111, ARS222, and BPR353 integration scheme with several spatial and temporal resolutions along the diagonal of the bar geometry.

As a first test we did the benchmark for electrophysiology with the cardiac monodomain equations as described in Niederer et al. (2011), with the suggested spatial resolutions of 0.5, 0.2, and 0.1 mm (using linear tetrahedral elements) and temporal resolutions of 0.05, 0.01, and 0.005 ms. We did the benchmark of propagation in a 3D slab with three different integration schemes: with FBE111 (forward-backward Euler), the ARS222 (Ascher et al., 1997), and the BPR353 schemes (Boscarino et al., 2013) (the numbers in the names of these integration scheme names reflect the number of explicit and implicit stages and the order of accuracy). As an extra, we also ran the benchmark using a large time step of 0.5 ms at a spatial resolution of 0.1 mm, to showcase the stability and temporal convergence of the used methods. The internal variables were stored at the quadrature points. In Figure 1 we display the activation times along the diagonal of the bar geometry. We see that increasing spatial and temporal resolutions have opposite effects on arrival times: increasing spatial resolution raises the arrival time, while increasing the temporal resolution lowers the arrival time. The faster convergence rate of the arrival time for higher order time integration is also noticeable. For example, for the BPR353 scheme the arrival times for the time steps of 0.05, 0.01, and 0.005 are almost indistinguishable. In Niederer et al. (2011) different codes were found to have arrival times between 37.8 and 48.7 ms at the highest spatial and temporal resolutions. Our arrival times are within those bounds at these highest resolutions. (It is inevitable that at lower resolutions the arrival time will deviate more.) Execution times for the simulations can be found in Table 1.

**Table 1:** Execution times for Niederer's electrophysiology benchmark.

**Scheme**	**Δx (mm)**	**Δt (ms)**	**Execution time (s)**
FBE111	0.1	0.5	4.20·10^3^
FBE111	0.1	0.05	1.27·10^4^
FBE111	0.1	0.01	6.34·10^4^
FBE111	0.1	0.005	1.24·10^5^
FBE111	0.2	0.05	1.81·10^3^
FBE111	0.2	0.01	9.00·10^3^
FBE111	0.2	0.005	1.82·10^4^
FBE111	0.5	0.05	2.94·10^2^
FBE111	0.5	0.01	1.48·10^3^
FBE111	0.5	0.005	3.10·10^3^
ARS222	0.1	0.5	5.40·10^3^
ARS222	0.1	0.05	2.51·10^4^
ARS222	0.1	0.01	1.25·10^5^
ARS222	0.1	0.005	2.48·10^5^
ARS222	0.2	0.05	3.48·10^3^
ARS222	0.2	0.01	1.77·10^4^
ARS222	0.2	0.005	3.44·10^4^
ARS222	0.5	0.05	4.30·10^2^
ARS222	0.5	0.01	2.19·10^3^
ARS222	0.5	0.005	4.40·10^3^
BPR353	0.1	0.5	1.10·10^4^
BPR353	0.1	0.05	5.27·10^4^
BPR353	0.1	0.01	2.59·10^5^
BPR353	0.1	0.005	5.22·10^5^
BPR353	0.2	0.05	7.28·10^3^
BPR353	0.2	0.01	3.57·10^4^
BPR353	0.2	0.005	7.12·10^4^
BPR353	0.5	0.05	7.45·10^2^
BPR353	0.5	0.01	3.67·10^3^
BPR353	0.5	0.005	7.15·10^3^

*Simulations were run on 32 nodes of Intel E5-2670 CPUs, using 1 core per node. See section 6.1 for details*.

### 6.2. Electromechanics

**Figure 2:**
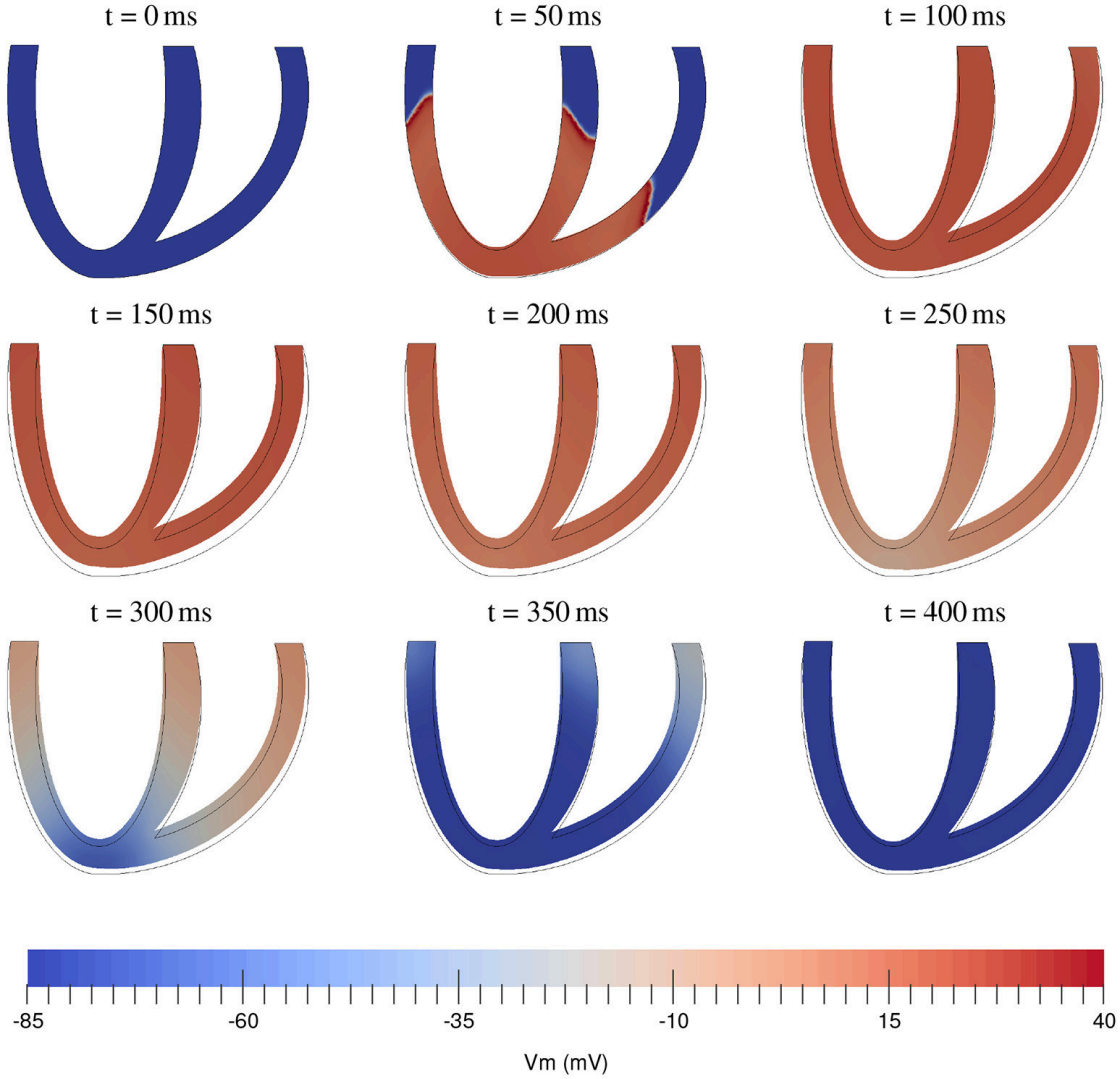
Time sequence of electromechanical contraction of a full 3D biventricular cardiac geometry. Color coding shows transmembrane potential.

At this stage of development of our package we decided just to illustrate the solution of electromechanical equations using the most simple tools. The comparison of various integration methods and constitutive relations will be done at a later stage. As an illustration for the fully coupled electromechanical equations we simulated the contraction of an idealized biventricular geometry that was stimulated at the apex. The mesh for this geometry was created using Gmsh (Geuzaine and Remacle, 2009) with a resolution of 0.2 mm resulting in a tetrahedral mesh consisting of 1529230 cells and 312888 vertices. We used the algorithm from Bayer et al. (2012) to generate myofiber orientations. The fiber angle varied from −45° (epi) to 75° (endo). We used the monodomain formulation and the TNNP06 (ten Tusscher and Panfilov, 2006) model for electrophysiology, with the same parameters as in Niederer et al. (2011). For the passive hyperelastic equations we used the Guccione constitutive equations (Guccione et al., 1995), where a penalty term κ/2(*J*−1)^2^ was added to the strain energy and for the active tension generation we used the Niederer-Hunter-Smith model (Niederer et al., 2006). Parameters were taken from Keldermann et al. (2010) and κ was taken as 350 kPa. Here we used a timestep of 0.5 ms with the FBE111 scheme and we used linear elements for the transmembrane voltage and deformations, while the internal variables were stored at the quadrature points. The resulting activation and contraction sequence can be seen in Figure 2. The simulation took 7.5 h on 32 nodes of Intel E5-2670 CPUs, using 1 core per node. The electromechanical testing will be continued in subsequent studies.

## 7. Discussion and outlook

In this paper we presented an overview of the methodology used in cardiac electromechanics and our numerical approach to these challenging problems. In particular, in section 2 we presented a short derivation of the main equations of electromechanics from basic principles (i.e., geometry and balance equations) in strong and weak form. We discussed constitutive equations to close these equations and clearly list all assumptions made. We derived a general representation of a deformation-dependent conduction tensor, assuming orthotropic symmetry and pointwise dependence on deformation and showed that previous deformation-dependent conduction tensors found in literature are all special cases of this. Note however, that the scalar functions in this representation still need to be determined from experiment. In section 3 we applied standard finite element methods to express the variational equations in a finite basis, which can then be solved by the numerical methods in section 4. There we discussed additive implicit-explicit Runge-Kutta time integration methods and how with appropriate partitioning of fast and slow physics the non-linear implicit equations can be solved more easily by solving smaller problems belonging to different fields one after another. Efficient (non-)linear solvers for these problems were also discussed. Further we reviewed the structure and possibilities of the GEMS library in section 5 and how PETSc gives us a wide range of tools to solve our PDE's, including meshes, I/O and solvers. In section 6 we presented some numerical results as verification and illustration of the GEMS library.

Our main conclusion is that additive implicit-explicit Runge-Kutta time integration methods, combining the advantages of implicit and explicit integration, work very well for electromechanical problems. This method allows larger time steps, with limited complication of Jacobians and non-linear solves. Our numerical implementation uses the PETSc library extensively, which gave us access to powerful and scalable mesh management, time stepping and (non)linear solvers which may need mesh and field information. One of the things which could be further researched is whether we can get much advantage of anistropic mesh adaptation through the PRAgMaTIc library (Rokos and Gorman, 2013), which has been recently interfaced to PETSc (Barral et al., 2016). This could also be used to build mesh hierarchies in a geometric multigrid approach.

The GEMS package is still in the process of further development. Although the user can access and set all solver options through the command line, a graphical user interface may be desirable in the future, both for input and visualization. Regarding modeling, we currently hard-coded two cell models (FHN and TP06) and foresee to import more models of cardiac electrophysiology in a semi-automated way via the CellML repository (www.cellml.org). We are currently using pressure boundary conditions on the endocardial surface, which can be extended with physical models for circulation and valve action.

Our method has been designed to enable strong coupling between the electrical and mechanical subsystems at every time step of the simulation, and at the same high spatial resolution, both for the electrical and mechanical equations. One possible speed-up factor is the following: currently all field values and gradients at the quadrature points are calculated for each residual or Jacobian belonging to some field(s), for maximum flexibility. Thus, one may avoid unnecessary interpolation in order to accelerate the computation of the residuals: if the residual of field A is independent of field B, the value or gradient of field B at the quadrature points is not needed.

The use of PETSc enables to parallelize the computation on high-performance computing clusters (HPC). Smaller (test) runs, can be run on a desktop computer, requiring about 32GB of RAM memory to run the biventricular model in Figure 2 with the TP06 cell model. There is no significant difference in memory cost between mono- and bidomain equations, since the latter introduces only few new state variables (extracellular potential, extracellular conductivities).

In this paper we have chosen to illustrate our approach using simple standard problems: the benchmark for electrophysiology (Niederer et al., 2011) and simple illustration of electromechanics for the fully coupled equations an idealized biventricular geometry. This is because we mainly wanted to describe of the methodology and place it to the existing environment and did not focus on specific scientific applications. Such simulations can definitely be performed using our methodology and will be presented in subsequent papers.

## Author contributions

AP and HD designed the research. SA implemented the methods. SA, HD, and AP wrote the manuscript.

### Conflict of interest statement

The authors declare that the research was conducted in the absence of any commercial or financial relationships that could be construed as a potential conflict of interest.

## Figures and Tables

**Table:** 

*A*	acceleration
*B*	body force
B	material manifold
*C*	deformation tensor
*E*	strain tensor
{_*E*_*A*_}*A*∈{*F, S, N*}_	material fiber directions
*F*	deformation gradient
*G*	material metric
*g*	spatial metric
*J*	Jacobian of deformation
*P*	first Piola-Kirchhoff stress tensor
*P*_*appl*_	applied pressure
*R*	reaction rates for internal variables
*S*	second Piola-Kirchhoff stress tensor
S	spatial manifold
Σ_*e*_	extracellular conduction tensor
Σ_*i*_	intracellular conduction tensor
*V*	velocity
*V*_*e*_	extracellular voltage
*V*_*m*_	transmembrane voltage
*X*	material coordinates
*x*	spatial coordinates
Γ	internal variables (i.e., ionic concentrations, gating variables, tensions variables)
ϕ	deformation field
